# A Novel MEMS Gyro North Finder Design Based on the Rotation Modulation Technique

**DOI:** 10.3390/s17050973

**Published:** 2017-04-28

**Authors:** Yongjian Zhang, Bin Zhou, Mingliang Song, Bo Hou, Haifeng Xing, Rong Zhang

**Affiliations:** Engineering Research Center for Navigation Technology, Department of Precision Instrument, Tsinghua University, Beijing 100084, China; zhangyongjian12@mails.tsinghua.edu.cn (Y.Z.); songml12@mails.tsinghua.edu.cn (M.S.); houb15@mails.tsinghua.edu.cn (B.H.); xhf15@mails.tsinghua.edu.cn (H.X.)

**Keywords:** MEMS gyro north finder, north finding, rotation modulation, robust least square method, robust Kalman filter

## Abstract

Gyro north finders have been widely used in maneuvering weapon orientation, oil drilling and other areas. This paper proposes a novel Micro-Electro-Mechanical System (MEMS) gyroscope north finder based on the rotation modulation (RM) technique. Two rotation modulation modes (static and dynamic modulation) are applied. Compared to the traditional gyro north finders, only one single MEMS gyroscope and one MEMS accelerometer are needed, reducing the total cost since high-precision gyroscopes and accelerometers are the most expensive components in gyro north finders. To reduce the volume and enhance the reliability, wireless power and wireless data transmission technique are introduced into the rotation modulation system for the first time. To enhance the system robustness, the robust least square method (RLSM) and robust Kalman filter (RKF) are applied in the static and dynamic north finding methods, respectively. Experimental characterization resulted in a static accuracy of 0.66° and a dynamic repeatability accuracy of 1°, respectively, confirming the excellent potential of the novel north finding system. The proposed single gyro and single accelerometer north finding scheme is universal, and can be an important reference to both scientific research and industrial applications.

## 1. Introduction

North finders have been widely used in many military areas such as tactical missile launchers, soldier navigation systems, and many other commercial areas such as coal mining, automatic driving systems, etc. The common north finders are mainly astronomical instruments, digital magnetic compasses (DMCs), dual-GPS north finders, angular molecular electronic transduction (MET) sensor-based north finders and gyroscope north finders. Astronomical instruments are very old and vulnerable to weather interference. DMCs are simple but the accuracy is usually low because the geomagnetic field is susceptible to ferromagnetic material interference [1]. A dual-GPS north finder whose antennas are separated by 3 m can reach 2-mil north finding precision [2], but that is too large and subject to the availability of GPS signals. Angular MET sensors are known for their low-noise performance and high sensitivity [3]. North finding with MET sensors is a quite attractive technique. It is based on the Coriolis principle, measuring the Coriolis acceleration caused by the rotating speed coupled with the horizontal component of the Earth’s angular velocity. The requirement of a high and rather steady speed makes it a challenge for the man-portable north finders [4].

In contrast, the gyro north finders are self-contained and cannot be affected by external interference such as weather and magnetic interference, so they have been widely used especially in the military field. Inertial-based sensors are found to be the most robust and reliable for north finding. The traditional gyro north finders often use high-precision gyroscopes, such as mechanical gyroscopes, ring laser gyroscopes (RLG) [5,6], fiber optical gyroscopes (FOG) [7,8], dynamically-tuned gyroscopes (DTG) [9], and hemispherical resonator gyroscopes (HRG) [10]. They can achieve high accuracy (1–2 mils or sub mils). However, they are mostly heavy (5 to 25 kg), high power consumption (tens of watts), bulky (a few tens of centimeters square) and quite expensive (several $10,000) [11]. Their high cost, large volume and high power consumption limit their applications in individual navigation and other fields. Current gyro north finders usually use an inertial measurement unit (IMU, three gyros and three accelerometers) to find north [5,10]. Some authors improved the use of two gyros and two accelerometers [12] or a single gyro and two accelerometers [7,9]. For practical applications, the dynamic range of the north finders can be small and its attitudes change very slowly. Its attitudes can be measured through rotating a single accelerometer. Therefore, a single gyro and a single accelerometer can be the minimum configuration for a gyro north finder.

MEMS gyros have the advantages of low cost, small size, low power, and high robustness compared to the traditional gyros. MEMS gyro north seeking is an attractive possibility, but MEMS gyros are yet to establish credibility in this high-precision domain [13]. MEMS gyro north seeking still faces with many practical problems, such as the turn-on to turn-on bias variability, bias drift, low signal to noise ratio (since the Earth’s rotation rate is a tiny value), lack of modern adaptive methods for azimuth error reduce for the MEMS gyros, and other problems.

A systematic error auto-compensation method, namely the rotation modulation technique, is analyzed and verified to help solve the above problems. Rotation modulation is often used to improve the inertial navigation system precision [14,15,16]. It refers to rotating the IMUs around one axis, or multiple axes continuously or discretely. The inertial sensors’ bias drift and other slowly changing errors can be modulated to zero-mean periodical values [14,16]. This makes the MEMS gyro north finder an intriguing possibility and a development trend [15]. Iozan and Zhang proposed to use MEMS gyros to measure the Earth’s rotation rate to verify its potential in north seeking [17,18]. Prikhodko analyzed the principle of MEMS gyro compassing, and implemented the rate table experiment of discrete turning and carouseling [13,19]. Johnson reported their latest MEMS gyroscope with Angle Random Walk (ARW) of 0.0019°/√hr and indexed the inertial sensor assembly (ISA) to verify its north-seeking ability [20,21]. However, the above studies either used rate table for verification or indexed (±180° turning) the ISA to find the north direction. Few works present a complete MEMS gyro north finder, let alone proved the design details of a practical MEMS gyro north finder.

The traditional north-seeking scheme is often implemented using the least square method (LSM) or Kalman filters because they are effective and easy to realize. The LSM and traditional Kalman filters use all of the observation values even they have abnormal values. The outliers affect the properties of Kalman filter innovation. This will introduce large errors. The robust methods are used to solve the problem [22]. The observation values are divided into three portions: complete trust, complete rejection and partial trust areas according to the observation variance [23]. Different weights are given by partitions, and then the robustness of the system can be improved. For increasing the robustness of the system, the robust least square method (RLSM) and robust Kalman filter (RKF) were introduced and implemented in the north-finding prototype.

In this paper, the RM technique is applied to use the MEMS gyros in the north finders. A novel MEMS gyro north finder based on RM is designed and implemented with the following three characteristics: First, the north finder is composed of only one single MEMS gyro and one single accelerometer. The cost and power consumption of the system are greatly reduced. Second, wireless power and wireless data transmission technique are introduced into the system so that the system could rotate discretely or continuously. It is the first time that these techniques have been integrated in the rotation modulation system. The volume and weight of the system are significantly decreased. Third, for practical application, the robust algorithms (including static RLSM and dynamic RKF) are implemented. Static and dynamic experimental results verify the feasibility of MEMS gyros in north finding. This scheme is universal for any kind of gyros, and can be an important reference to both scientific research and industrial applications.

The rest of this paper is organized as follows: The rotation modulation principle, and the all-attitude static and dynamic gyro north finding models are analyzed in Section 2. Design and implementation of the prototype are presented in Section 3. The static and dynamic north finding results and error analysis of the prototype are given in Section 4, followed by a brief conclusion in Section 5.

## 2. Principle and Theoretical Model

### 2.1. Rotation Modulation Principle

**Figure 1:**
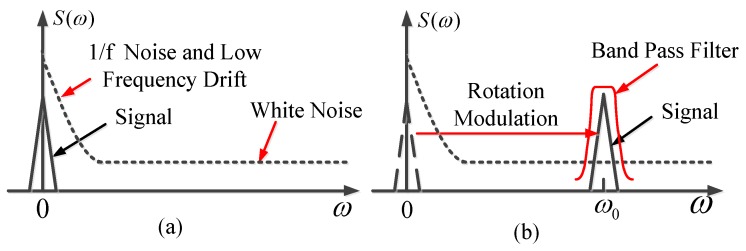
Rotational modulation principle in the frequency domain: (**a**) without rotation modulation (RM); (**b**) with RM.

The rotation modulation (RM) technique was first introduced in the 1980s for marine inertial navigation systems [24]. It has also been studied in the low-cost MEMS IMUs in recent years [14,25]. From the frequency domain, there is a more intuitive understanding of the rotation modulation. 1/f flicker noise and low-frequency drift are two of the main errors for low-cost inertial devices [16]. To find the north, it is essential to measure the Earth’s rotation rate, which is a small DC constant in the frequency domain. The signal is submerged in the high-amplitude noise. It is difficult to directly extract the signal from the noise using the general signal processing method as shown in Figure 1. Rotation modulation uses the physical rotation to modulate the signal to the rotation frequency point, then improves the signal-to-noise ratio by separating the signal with the low-frequency noise. Other methods such as a band pass filter or demodulation are used to obtain the original signals. The rotation modulation technique can operate in two modes: discrete rotation modulation (static modulation) and continuous rotation modulation (dynamic modulation).

### 2.2. Static North Finding Model

**Figure 2:**
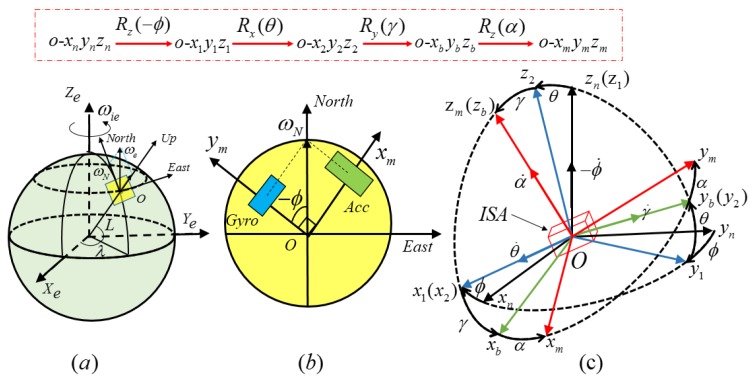
(**a**) The coordinate frames definitions, (**b**) the heading angle definition in the *n*-frame (**c**) and the transformation process between coordinate frames.

The static north finding is based on the principle of discrete rotation modulation. The coordinate frames are defined as follows: the traditional Earth-centered Earth-fixed frame, *e*-frame; the gyros and accelerometers consist of the measurement coordinate frame, *m*-frame; the north finder coordinate frame denotes as the body frame, *b*-frame; and the navigation coordinate frame, *n*-frame, is the ideal local level ENU (East-North-Up) geodetic frame. The coordinate frames definitions are illustrated in Figure 2a. In Figure 2a, *L* and *λ* represent the latitude and longitude of the Earth, *ω_ie_* and *ω_N_* represent the Earth’s rotation rate and its north projection on the *n*-frame. Acc is short for accelerometer and Gyro for gyroscope. ISA is the abbreviation for inertial sensor assembly.

The *b*-frame can be obtained from three Euler angle transformation (i.e., heading angle  ϕ, pitch angle *θ*, and roll angle *γ*, successively) from the *n*-frame. The heading angle is defined as [0, 360), which is measured clockwise from north. The pitch angle and roll angle are defined as [−90, 90], which follow the right-hand rule. The *m*-frame can be obtained by rotating the angle *α* around the *Z*-axis of the *b*-frame. The transformation process is illustrated in Figure 2c. The heading angle is denoted as (−ϕ) to match the right-hand rule in Figure 2b.

In Figure 2, $R_{x}\left(\theta \right)$ denotes rotating *θ* degrees around the *X* axis, $R_{y}\left(\gamma \right)$ denotes rotating *γ* degrees around the *Y* axis, and $R_{z}\left(-\phi \right)$ denotes rotating ϕ degrees around the *Z* axis reversely. Then the direction cosine matrix (DCM) from the *n*-frame to the *b*-frame can be obtained as follows:

(1) $$C_{n}^{b}=C_{2}^{b}C_{1}^{2}C_{n}^{1}$$

The above DCMs are defined as follows:

(2) $$C_{n}^{1}=R_{z}\left(-\phi \right)=\left[\begin{array}{ccc} \cos\phi & \sin\left(-\phi \right) & 0\\ -\sin\left(-\phi \right) & \cos\phi & 0\\ 0 & 0 & 1 \end{array}\right]$$

(3) $$C_{1}^{2}=R_{x}\left(\theta \right)=\left[\begin{array}{ccc} 1 & 0 & 0\\ 0 & \cos\theta & \sin\theta \\ 0 & -\sin\theta & \cos\theta \end{array}\right]$$

(4) $$C_{2}^{b}=R_{y}\left(\gamma \right)=\left[\begin{array}{ccc} \cos\gamma & 0 & -\sin\gamma \\ 0 & 1 & 0\\ \sin\gamma & 0 & \cos\gamma \; \end{array}\right]$$

The DCM from the *b*-frame to the *m*-frame is expressed as:

(5) $$C_{b}^{m}=R_{z}\left(\alpha \right)=\left[\begin{array}{ccc} \cos\alpha & \sin\alpha & 0\\ -\sin\alpha & \cos\alpha & 0\\ 0 & 0 & 1 \end{array}\right]$$

The Earth’s rotation rate $\omega_{ie}\;$ is 15.0411°/h. Its projection in the *n*-frame is expressed as:

(6) $$\omega_{ie}^{n}={\left[\begin{array}{ccc} 0 & \omega_{ie}\cos L & \omega_{ie}\sin L \end{array}\right]}^{T}$$

The projection of the Earth’s gravitational acceleration in the *n*-frame is shown as follows:

(7) $$f_{n}={\left[\begin{array}{ccc} 0 & 0 & -g \end{array}\right]}^{T}$$

Then the outputs of the gyro in the *m*-frame is calculated as:

(8) $$W_{iem}=C_{n}^{m}\omega_{ie}^{n}=C_{b}^{m}C_{n}^{b}\omega_{ie}^{n}$$

From above formula, the outputs of the three axial gyroscopes in the *m*-frame are obtained:

(9) $$W_{iem}=\left[\begin{array}{c} W_{iemx}\\ W_{iemy}\\ W_{iemz} \end{array}\right]=\left[\begin{array}{c} \omega_{ie}\sin L\left(-\cos\alpha \cos\theta \sin\gamma +\sin\alpha \sin\theta \right)+\omega_{ie}\cos L\left(\cos\theta \cos\phi \sin\alpha +\cos\alpha \left(\cos\phi \sin\gamma \sin\theta -\cos\gamma \sin\phi \right)\right)\\ \omega_{ie}\sin L\left(\cos\theta \sin\alpha \sin\gamma +\cos\alpha \sin\theta \right)+\omega_{ie}\cos L\left(\cos\alpha \cos\theta \cos\phi -\sin\alpha \left(\cos\phi \sin\gamma \sin\theta -\cos\gamma \sin\phi \right)\right)\\ \omega_{ie}\sin L\cos\gamma \cos\theta +\omega_{ie}\cos L\left(-\cos\gamma \cos\phi \sin\theta -\sin\gamma \sin\phi \right) \end{array}\right]$$

Similar to the gyroscope, the accelerometer’s outputs in the *m*-frame are expressed as:

(10) $$f_{m}=\left[\begin{array}{c} f_{mx}\\ f_{my}\\ f_{mz} \end{array}\right]=C_{n}^{m}f_{n}=C_{b}^{m}C_{n}^{b}f_{n}=\left[\begin{array}{c} g\left(\cos\alpha \cos\theta \sin\gamma -\sin\alpha \sin\theta \right)\\ -g\left(\cos\theta \sin\alpha \sin\gamma +\cos\alpha \sin\theta \right)\\ -gcos\gamma cos\theta \; \end{array}\right]$$

For the traditional gyro north finder,$\;W_{iemx}$, $W_{iemy}$ and $f_{mx}$,$\;f_{my}$ are used to calculate the heading angle. This is the two-gyros and two-accelerometers north finding scheme. If only $\;W_{iemx}$ or $W_{iemy}$ are used, then it is the scheme of one gyro and two accelerometers. In this paper, a single gyroscope, single accelerometer north finding scheme is proposed. This inertial sensor configuration can reduce the system cost as well as system volume. On the other hand, if two or more gyros and accelerometers are used with our scheme for redundant configuration, the required alignment time can be reduced or the system accuracy can be improved.

The MEMS accelerometer is installed on the *X* axis, and the gyro on the *Y* axis of the *m*-frame. Considering the gyro’s bias $\;E_{b}$, the gyro’s output is expressed as:

(11) $$W_{iemy}=\omega_{ie}*\sin\alpha \left(\sin L\cos\theta \sin\gamma -\cos L\cos\phi \sin\gamma \sin\theta +\cos L\cos\gamma \sin\phi \right)+\omega_{ie}*\cos\alpha \left(\sin L\sin\theta +\cos L\cos\theta \cos\phi \right)+E_{b}$$

where $W_{iemy}$ represents the gyro’s output. To simplify the above formula, *A* and *B* are used:

(12) $$\{\begin{array}{l} A=\omega_{ie}\left(\sin L\cos\theta \sin\gamma -\cos L\cos\phi \sin\gamma \sin\theta +\cos L\cos\gamma \sin\phi \right)\\ B=\omega_{ie}\left(\sin L\sin\theta +\cos L\cos\theta \cos\phi \right) \end{array}$$

From the above Equations (11) and (12), we can see that the modulated gyroscope output is relevant to the earth rotation rate. The amplitude of the modulated signal can be used as a good verification of true north finding. Especially when the pitch angle and roll angle are zero, the gyro output can be simplified as follows:

(13) $$W_{iemy}=\omega_{ie}\cos L\sin\alpha \sin\phi +\omega_{ie}\cos L\cos\alpha \cos\phi +E_{b}=\omega_{ie}cosL\cos\left(\alpha -\phi \right)+E_{b}$$

From the above expression, we can see that the amplitude of the modulated signal equals to the projection of the Earth’s rotation rate on the local level, which is *ω_ie_* × cos*L*. For our experiments conducted in Beijing, the latitude is 40 degrees. The amplitude of the modulated gyro output should be around 0.0032°/s.

Similar to the gyroscope, the accelerometer in the *X* axis is expressed as follows:

(14) $$f_{mx}=g\cos\alpha \cos\theta \sin\gamma -g\sin\alpha \sin\theta +E_{a}$$

where $f_{mx}$ represents the accelerometer’s output, $E_{a}$ is the accelerometer’s bias. *C* and *D* are used:

(15) $$\{\begin{array}{l} C=-gsin\theta \\ D=gcos\theta sin\gamma \; \end{array}$$

Then Equations (11) and (14) can be rewritten as follows:

(16) $$\{\begin{array}{l} W_{iemy}=A*sin\alpha +B*cos\alpha +E_{b}\\ f_{mx}=C*sin\alpha +D*cos\alpha +E_{a} \end{array}$$

For the static north finding model, the gyroscope and the accelerometer are rotated in different directions. For the 36-point static north finding, the accelerometer’s outputs are expressed as:

(17) $$\{\begin{array}{c} f_{mx1}=C*sin\alpha_{1}+D*cos\alpha_{1}+E_{a}\\ f_{mx2}=C*sin\alpha_{2}+D*cos\alpha_{2}+E_{a}\\ \ldots \\ f_{mx36}=C*sin\alpha_{36}+D*cos\alpha_{36}+E_{a} \end{array}$$

Denote the above symbols as:

(18) $$Z=\left[\begin{array}{c} f_{mx1}\\ f_{mx2}\\ \ldots \\ f_{mx36} \end{array}\right],H=\left[\begin{array}{c} \begin{array}{ccc} \sin\alpha_{1} & \cos\alpha_{1} & 1 \end{array}\\ \begin{array}{ccc} \sin\alpha_{2} & \cos\alpha_{2} & 1 \end{array}\\ \ldots \\ \begin{array}{ccc} \;\sin\alpha_{36} & \cos\alpha_{36} & 1 \end{array} \end{array}\right],X=\left[\begin{array}{c} C\\ D\\ E_{a} \end{array}\right]$$

In matrix form, Equation (16) above can be written as

(19) $$Z=\left[\begin{array}{c} f_{mx1}\\ f_{mx2}\\ \ldots \\ f_{mx36} \end{array}\right]=\left[\begin{array}{c} \begin{array}{ccc} \sin\alpha_{1} & \cos\alpha_{1} & 1 \end{array}\\ \begin{array}{ccc} \sin\alpha_{2} & \cos\alpha_{2} & 1 \end{array}\\ \ldots \\ \begin{array}{ccc} \sin\alpha_{36} & \cos\alpha_{36} & 1 \end{array} \end{array}\right]\left[\begin{array}{c} C\\ D\\ E_{a} \end{array}\right]=HX$$

The *X* value can be obtained by the Least Square Method (LSM) as follows:

(20) $$\hat{X}={\left(H^{T}H\right)}^{-1}H^{T}Z$$

The value of *C* and *D* can be obtained, then the pitch angle *θ* and roll angle *γ* can be calculated through Equation (14). Applying the LSM algorithm to the gyroscope’s output, the values of *A* and *B* can be obtained. Then the heading angle ϕ can be calculated as follows:

(21) $$\tan\phi =\frac{\sin\phi }{\cos\phi }=\frac{\left(\frac{A}{\omega_{ie}}-\sin L\cos\theta \sin\gamma +\cos L\cos\phi \sin\gamma \sin\theta \right)/\cos\gamma }{\left(\frac{B}{\omega_{ie}}-\sin L\sin\theta \right)/\cos\theta }$$

The above derivation of the static multi-position north finding scheme uses the outputs of the gyro and accelerometer without distinction. Actually, the outputs of the gyro and accelerometer are not always accurate. The outputs are often contaminated by noise from the platform jitter or personnel movement. The abnormal values or outliers are also inevitable. Thus, the RLSM was designed to tackle this issue. The basic principle of the RLSM is to make full use of the effective information, limit the use of reduced-effectiveness information and eliminate the harmful information.

The traditional weighted LSM can be derived from the traditional LSM (Equation (20)) using the weight function *W*. Then the solution of *X* can be expressed as follows:

(22) $$\hat{X}={\left(H^{T}WH\right)}^{-1}H^{T}WZ$$

The weight function can be given according to the standard deviation of the measurements. The IGG-III criterion weight function is often used [23]:

(23) $$w_{j}=\{\begin{array}{cc} 1 & ,\left|u_{j}\right|<k_{0}\\ \frac{k_{0}}{\left|u_{j}\right|}{\left(\frac{k_{1}-\left|u_{j}\right|}{k_{1}-k_{0}}\right)}^{2} & ,k_{0}\leq \left|u_{j}\right|<k_{1}\\ 0 & ,k_{1}\leq \left|u_{j}\right| \end{array}$$

where $u_{j}$ is the standardized residuals, $w_{j}$ is the weight of the *j*-th observations, $k_{0}$ and $k_{1}$ are the critical values of the weight function, $k_{0}$ is 1–1.5, and $k_{1}$ is 2.5–8.

For the given weight function, the algorithm is robust to the outliers and abnormal values. The values of *A*, *B*, *C*, and *D* can be calculated. Then the heading angle can be obtained. This is the RLSM scheme for the static single gyro north finder.

### 2.3. Dynamic North Finding Model

The dynamic north finding is based on the principle of continuous rotation modulation. A Kalman filter can realize real-time and effective estimation of the system parameters, and is widely used in many fields, especially in the field of inertial navigation system. A robust Kalman filter is used for dynamic north finding. For Equation (15), choose *A*, *B*, *C*, *D*, $\;E_{b}$ and $E_{a}$ as the system state, then the state vector can be expressed as:

(24) $$X={\left[\begin{array}{cc} A & \begin{array}{ccc} B & E_{b} & \begin{array}{ccc} C & D & E_{a} \end{array} \end{array} \end{array}\right]}^{T}$$

The system state equation is:

(25) $$X_{k+1}=X_{k}+W_{k+1}$$

where $W_{k+1}$ denotes the system process noise. Taking the outputs of the MEMS gyro and accelerometer as the observation, the system observation equation is established as follows:

(26) $$Z_{k}=\left[\begin{array}{c} W_{iemy}\\ f_{mx} \end{array}\right]=h_{k}X_{k}$$

For data observation, the dynamic north finding model uses the gyro and accelerometer outputs directly while the static method uses the averaged outputs in each direction.

(27) $$h_{k}=\left[\begin{array}{c} \sin\alpha_{k}\;\cos\alpha_{k}\;1\;0\;0\;0\\ \;0\;0\;0\;\sin\alpha_{k}\;\cos\alpha_{k}\;1 \end{array}\right]$$

where $h_{k}$ denotes the measurement matrix. The system state equation and the observation equation of the Kalman filter are shown above. The traditional Kalman updating equations can be used to solve the equations. For the dynamic north finding algorithm, the state vector *X* is calculated out in each Kalman updating cycle while *A*, *B* and *C*, *D* are calculated out in each accelerometer and gyro updating progress respectively for the static algorithm.

The outputs of the gyro and accelerometer are used as the measurements directly. It is easy to understand. However, the direct outputs of the gyro and accelerometer are susceptible to interference. Similar to the static north finding method, a RKF is used to improve the estimation robustness. The weight function G(k+1) is added after the Kalman gain $\;K_{k}$:

(28) $${\hat{X}}_{k}={\hat{X}}_{k/k-1}+K_{k}G\left(k+1\right)\left(Z_{k}-H_{k}{\hat{X}}_{k/k-1}\right)$$

The Chi-Square test was used for fault detection and estimation of outliers [26]. Then the weight  G(k+1) can be obtained by the threshold and the measurement standard deviation. The weight function can also use the IGG-III function or Huber function [23].

Similar with the static method, the pitch angle *θ* and roll angle *γ* can be obtained from *C* and *D*. With *A* and *B*, the heading angle can be calculated. For the dynamic north finding, the algorithm should be implemented in a microcontroller in real-time. Equation (21) should be simplified. When *γ* and *θ* are small angles, Equation (21) can be simplified as:

(29) $$\tan\phi =\frac{\sin\phi }{\cos\phi }=\frac{\frac{A}{\omega_{ie}}-\sin L\cos\theta \sin\gamma }{\frac{B}{\omega_{ie}}-\sin L\sin\theta \;}$$

Using the RKF north finding algorithm described above, the heading angle ϕ can be obtained.

## 3. Design and Implementation

### 3.1. Inertial Sensor Performance

For the proposed MEMS gyro north finder, its heading accuracy can be described as:

(30) $$\sigma_{heading}=\sqrt{\sigma_{gyro}^{2}+\sigma_{acc}^{2}+\sigma_{other}^{2}}$$

where $\sigma_{heading}$ denotes the heading accuracy, $\sigma_{gyro\;}\;\mathrm{and}\;\sigma_{acc}$ are the heading errors caused by the gyro and accelerometer, and $\sigma_{other}$ is the heading error caused by other sources. The gyro’s precision directly determines the accuracy of the gyro north finder. The gyro errors (including ARW, *g*-sensitivity) have big influence on the heading accuracy, they should be carefully analyzed and compensated. The accelerometer is used to compensate the attitude angle error. It is also a key component. For the tiny value of the Earth’s rotation rate, any other minor errors may cause measurement errors, such as mechanical interference, vortex motion [27], speed fluctuation, uncompleted tilt compensation. These errors should also be carefully handled to ensure that their impacts are in a certain amount level. For example, the mechanical structure needs to be carefully designed, precisely machined and assembled. The runout of the motor axis should also be minimized.

For static north finding scheme, the heading error caused by the gyro is mainly dependent on the latitude *L*, the required alignment time *Ta*, and the gyro’s ARW, as expressed in the following Equation [28]:

(31) $$\sigma_{gyro}=\frac{ARW}{\omega_{ie}*\cos L*\sqrt{T_{a}}}$$

The above equation can be used as a criterion for gyro selection. The gyro’s ARW requirement is related to the required alignment time *T_a_* and the heading accuracy $\;\sigma_{gyro}$, as well as the Latitude *L*. To achieve the heading accuracy of 1°, if the required alignment time is five minutes, the required gyro’s ARW should be better than 0.058°/√hr. The above discussion is the simplified analysis that only considered the gyro main error sources. In fact, to achieve the designed heading accuracy, the gyro g-sensitivity and temperature error should be considered. These errors will be discussed in Section 4.3.

For the dynamic north finding scheme, the influence of the gyro on north finding becomes complicated. The equivalent precision of the gyro drift can be used to estimate the north finding accuracy. Generally speaking, rotation modulation can improve the accuracy of the equivalent gyro drift 5–30 times [29].

The accelerometer is used to compensate the attitude error. The vertical component of the Earth’s angular velocity is introduced into the gyro’s output if there exists an accelerometer’s bias error [28]:

(32) $$\sigma_{acc}=\frac{\varepsilon_{E}}{\omega_{ie}*\cos L\;}=\frac{\omega_{ie}*\sin L\sin\beta }{\omega_{ie}*\cos L\;}=\frac{b_{acc}}{g}\tan L$$

where $\varepsilon_{E}$ is the equivalent east gyro drift, g is the gravity acceleration, $b_{acc}$ is the accelerometer’s bias, and β is the error angle produced by the accelerometer’s bias, where $\;b_{acc}=\mathrm{gsin}\beta$. Equation (32) can be used as a criterion for accelerometer selection. The accelerometer’s bias affects the accuracy of attitude angles. If the required heading accuracy $\sigma_{heading}\;$ is less than 1°, then the error caused by accelerometer had better be less than 0.05°. Then the bias requirement can be calculated using Equation (32), which is 1.04 mg. This accuracy requirement is easy to meet for the current commercial accelerometers on the market.

**Figure 3:**
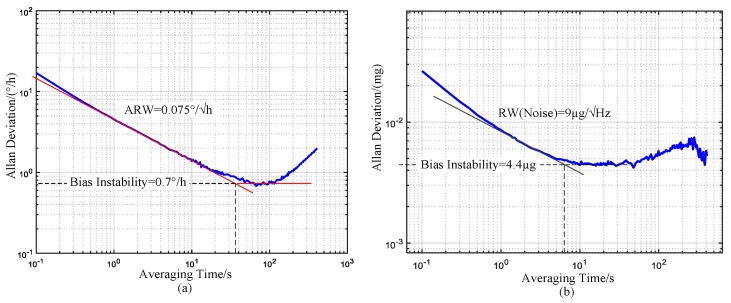
(**a**) Gyroscope Allan deviation; (**b**) Accelerometer Allan deviation.

In our prototype, a center support quadruple mass gyroscope (CSQMG) [30] made in-house and Colibrys MEMS accelerometer VS1002 were used. The measured Allan deviation curves of the MEMS gyro and accelerometer are displayed in Figure 3.

As shown in Figure 3, the gyro’s ARW is 0.075°/√hr. If the alignment time is one hour, the theoretical accuracy should be 0.66° in Beijing, where the latitude is 40 degrees. The gyro’s bias instability is around 0.7°/h. Through continuous rotation modulation, the equivalent gyro drift can be better than 0.2°/h, and a heading accuracy of better than 1° can be expected theoretically. The measured bias of the accelerometer is 0.23 mg. The north finding error caused by the accelerometer can be estimated using Equation (32). It is about 0.01° and can be ignored. The above discussion is the ideal estimation. It does not consider the gyro’s temperature drift error and other errors of the system. Those errors are discussed in Section 4.3.

### 3.2. Hardware Design

According to the mentioned north finding models, the MEMS gyro north finder prototype is designed and implemented. One uniaxial MEMS gyro and uniaxial accelerometer are used. To distinguish them from the traditional IMUs, the name Inertial Sensor Assembly (ISA) is used in our system.

To rotate discretely or continuously, a motor should be designed within the gyro north finder. The brushed DC motor has friction, while the brushless DC motor (BLDC) has a torque fluctuation, so a three-phase permanent magnet synchronous motor (PMSM) was chosen for the prototype. To realize continuous rotation, slip rings should be chosen for electrical connections, but the friction will also reduce the system’s performance and service life, and it will increase the system volume. On the other hand, wireless power and wireless data transmission only need to be roughly aligned as compared to the high-precision mounting requirements between the stator and rotor of slip rings, which decreases the system reliability. Therefore, the wireless power and wireless data transmission technique are introduced.

**Figure 4:**
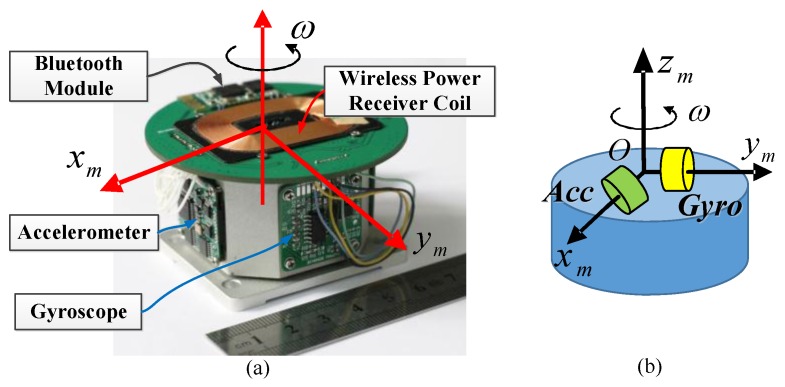
(**a**) Inertial sensor assembly (ISA) pictures; (**b**) ISA schematic diagram.

**Figure 5:**
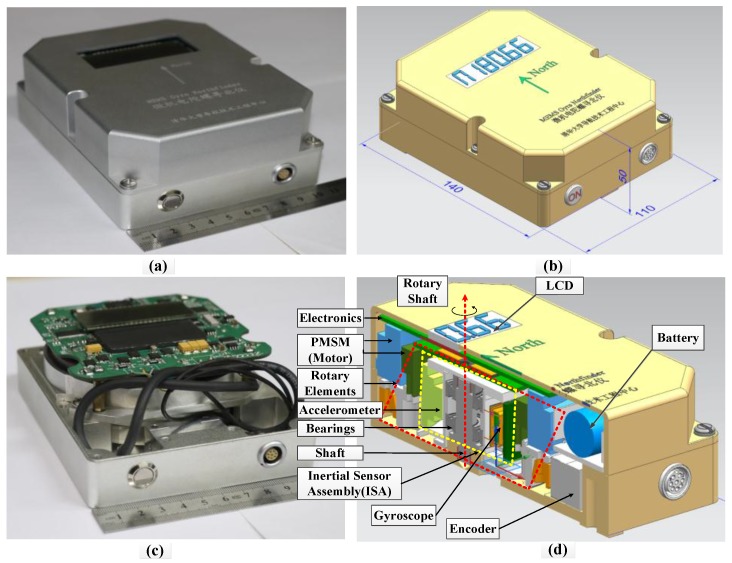
Structure pictures of MEMS gyro north finder prototype: (**a**) outline picture; (**b**) outline structure; (**c**) internal picture; (**d**) internal structure.

The MEMS gyro and accelerometer were installed along two axes perpendicular to each other within the same plane as shown in Figure 4. The Bluetooth module and the wireless power receiver were mounted on the top printed circuit board (PCB). The ISA was then fixed to the motor’s rotor, which was embedded in the north finder. In this way, the system size can be significantly reduced. The motor’s stator was also embedded inside the shell. Through ingenious design, the whole system is as small as a mobile hard disk. Figure 5 shows pictures of the structure of the MEMS gyro north finder prototype. To display the north finding results, a low-power liquid crystal display (LCD) was also implemented. The system size of the prototype is only 140 × 110 × 50 mm^3^. The weight is around 1.5 kg. The total power is about 3.6 W.

To seek north, the PMSM should be controlled to stay at a given position or rotate at a given speed. To realize this control, angular feedback was needed. A 100 mm absolute circular grating encoder and 32-bit BiSS protocol reading head from Renishaw were selected in our prototype. The resolution as stated in the Renishaw’s datasheet (No. L-9517-9399-01-D) is 0.0003″, but this resolution is below the noise floor of the encoder. The actual resolution is better than 0.3″. The system nominal accuracy including installation error is ±2.86″. The absolute angular feedback was also used in the static and dynamic north finding method.

### 3.3. Electronics Design

**Figure 6:**
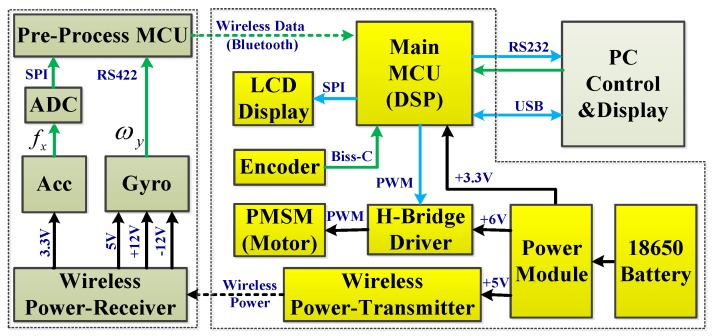
Electronics schematic of the north finder.

Figure 6 shows the electronics schematic of the north finder, which contains two parts: one part is the pre-process MCU (Micro-Controller Unit) board. Its main function is to collect the outputs of the gyro and accelerometer, preprocess the data (temperature compensation etc.), and send the data to the main MCU board via Bluetooth. To reduce the system power consumption, the TI low-power MCU MSP430 was chosen. The other part is the main MCU board. It is designed to receive the PC control commands, send the original data to the PC, implement the PMSM vector control algorithm, run the static and dynamic north finding algorithm, and display the results on the LCD. The real-time requirement is high, so a high-performance digital signal processor (DSP) was chosen. The specific DSP type is TI TMS320F28069. A 6 V DC power supply was used for the whole system.

A wireless power supply module, which could provide 5–10 W of wireless power was designed in the proposed system. Considering the requirement of data rate, power consumption and reliability, the Bluetooth was selected for wireless data transmission. A Bluetooth module named HC-05 was used in the system. The original sample rate is 200 Hz. The baud rate of the Bluetooth is 230,400 bps. Each data frame transmits 20 bytes of data (four bytes for system information, and eight bytes for the gyroscope and accelerometers, respectively).

### 3.4. PMSM Vector Control

To implement the discrete and continuous rotation modulation, one of the most important components is the drive unit. To rotate the ISA as smoothly as possible, a permanent magnet synchronous motor (PMSM) was chosen as the drive unit. Compared to the trapezoidal BEMF (back electromotive force) of the BLDC (brushless direct current motor), the BEMF of the PMSM is a sinusoidal wave. The rotor of PMSM is made of the permanent magnets. The stator of the PMSM is made of three-phase windings. To rotate the PMSM, three-phase sine wave currents are connected to the three windings. The three-phase currents have a phase difference of 120° from each other. According to the electromagnetic induction law, these three sine currents will produce a rotating magnetic field. The rotating magnetic field interacts with the motor rotor’s magnetic field to produce a rotational torque to drive the motor rotor.

**Figure 7:**
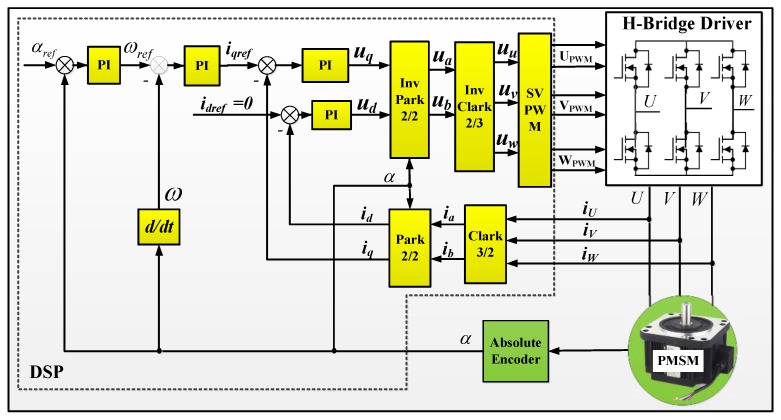
Schematic diagram of the permanent magnet synchronous motor (PMSM) vector control.

To produce a stable rotating magnetic field, some special control techniques such as direct torque control (DTC) or vector control (also called field-oriented control, FOC) are needed. To produce a smooth rotation, the PMSM vector control was chosen for our prototype. The schematic diagram of the PMSM vector control can be illustrated in Figure 7. *u* and *i* represent the voltage and current, respectively. *U*, *V* and *W* represent the three phase of the PMSM. The three phase currents *i_U,_ i_V_ and i_W_* of the PMSM are collected first, then transformed into the two phase current *i_a_* and *i_b__b_* in the motor stator *a*-*b*-frame. With the rotor rotating angle *α*, the two phase stator currents are transformed to the rotor *d*-*q*-frame. With the proper PI controller parameters, the direct-axis current *i_d_* can be controlled to zero. Then the torque can be proportional to the quadrature-axis current *i_q_*. The control of the PMSM becomes the same as a DC motor. More details about the vector control of PMSM can be found in our previous work [31].

### 3.5. Experiment Setup

**Figure 8:**
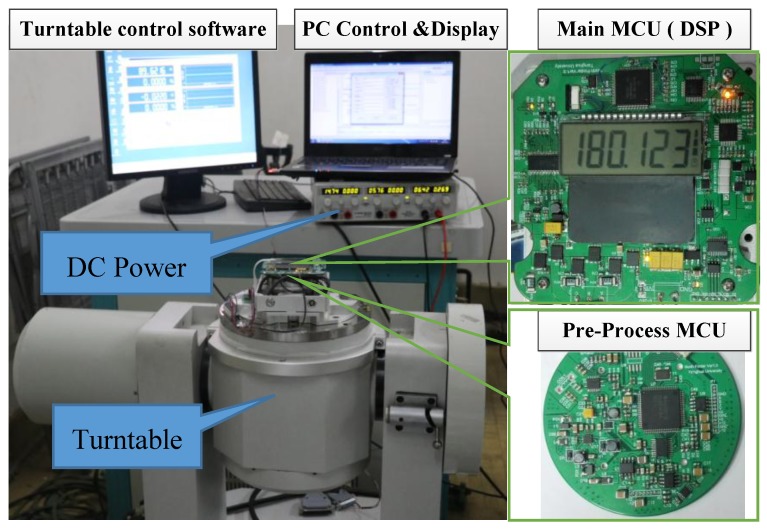
Experimental platform for testing the prototype, a high-precision turntable, a DC power source, the main MCU printed circuit board (PCB) and the pre-process MCU PCB of the prototype.

The experimental platform is shown in Figure 8. A turntable was used for the calibration of the gyroscope and the verification of the static and dynamic north finding experiments. A 6 V DC power source was used to supply the MEMS gyro north finder prototype. The normal current consumption is 0.5–0.6 A. The prototype was connected to a laptop computer through an RS232 interface. The commands were sent to the prototype and the data were sent to the computer.

## 4. Experimental Results and Error Analysis

### 4.1. Static North Finding

The previous work has shown that the position repeatability of the prototype can reach 0.0002° (peak-to-peak) [31]. The following experiments are carried out to verify the accuracy of static 36-position north finding algorithm. The north finder was pointed in one direction on the platform. Then the PMSM rotated with the ISA in given directions. The angle between two positions is 10° (360° for 36 positions). The time stayed at each direction is 30 s (including the rotating process). In order to eliminate the influence of the acceleration and deceleration of the motor during the rotating process, the gyro and accelerometer data during the rotation process were abandoned in data processing. A one-hour continuous experiment was conducted. The outputs of the encoder, gyro and accelerometer were collected at a sample rate of 200 Hz. Then the heading angle was calculated using the RLSM algorithm after every 36-point. The 85 (3600 s/30 s − 36 + 1 = 85) results for one-hour experiment were obtained recursively.

**Figure 9:**
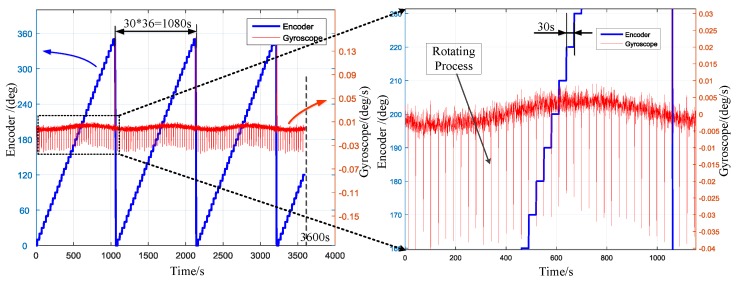
The outputs of the encoder and gyroscope (rate: 10 Hz).

**Figure 10:**
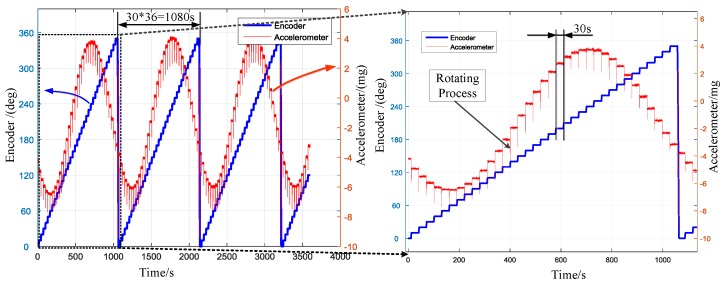
The outputs of encoder and accelerometer (rate: 10 Hz).

**Figure 11:**
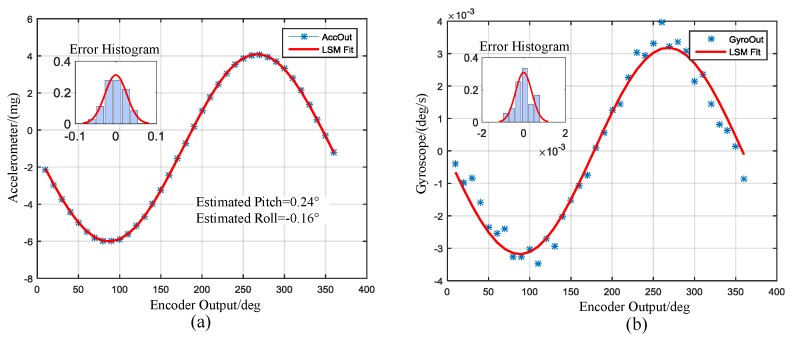
(**a**) Averaged outputs of the accelerometer and least square method (LSM) fit curve; (**b**) Averaged outputs of the gyroscope and LSM fit curve. Inset: the fitting error histogram of the LSM.

To facilitate observation and comparison, the raw outputs were resampled to 10 Hz (i.e., averaged every 20 samples). The outputs of the encoder and gyroscope are displayed in Figure 9. The left ordinate indicates the encoder, while the right ordinate indicates the gyro. The local enlarged image of a computational duration is presented in the right part of Figure 9. The sinusoid shape indicates the projection of the north component of the Earth’s rotation rate on the gyroscope input axis. Figure 10 shows the outputs of encoder and accelerometer. The sinusoid shape indicates the attitude angle of the prototype. The peaks indicate the rotating process as marked in the picture. The averaged outputs for one direction to be calculated and the LSM fitting curves are presented in Figure 11. Insets in Figure 11 are the fitting error histogram with normal distribution fits. The fit to the normal distribution curve revealed a Gaussian error model for the accelerometer and gyro data.

**Figure 12:**
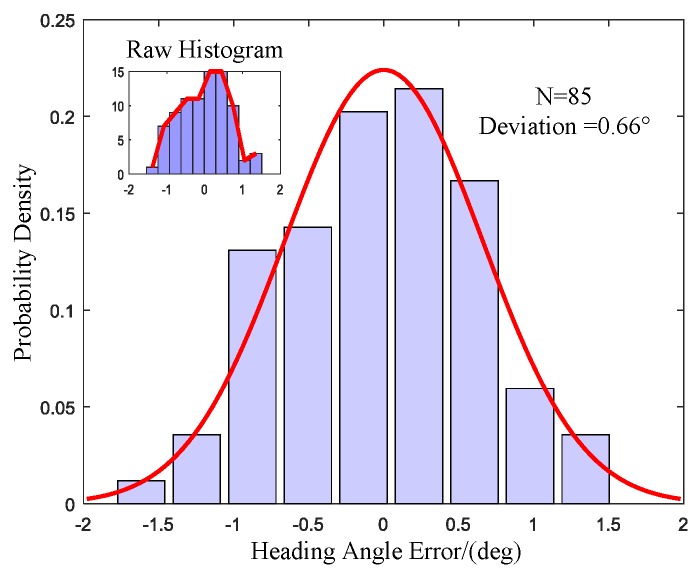
Heading angle error histogram with normal distribution fit, Inset: raw histogram.

The probability density histogram of the heading angle error is exhibited in Figure 12. The inset is the raw heading error histogram of the 85 north finding results. The error distribution was tested by the normal distribution. The shape is in good agreement with the normal distribution curve. The fit to the normal distribution curve revealed a Gaussian error model for the measurement errors. The deviation of 0.66° can be used to express the north finding accuracy. The experimental results are in good agreement with the theoretical analysis.

### 4.2. Dynamic North Finding

The dynamic north finding algorithm was verified on the turntable. As can be seen from the gyro Allan deviation in Figure 3a, the Allan deviation decreases with average time before 60 s, which means the error caused by gyro ARW decreases. Then the Allan deviation increases after 100 s, which indicates the gyro error caused by gyro RRW increases quickly. There is a clear correspondence between Allan deviation and power spectral density [32]. A rotation period of 60 s was chosen to make a balance between minimizing the north finding error and reducing the alignment time. Then the rotation speed was set to 6°/s for the dynamic north finding experiment.

To test the dynamic north finding accuracy, the prototype was set to a certain direction on the platform. The PMSM rotated continuously with the ISA at the speed of 6°/s. The speed stability is about 0.1%. The rotation duration was three minutes for each experiment. To test the repeatability of the prototype, seven independent experiments were performed. The original sample rate was 200 Hz, then resampled to 10 Hz. The proposed robust Kalman filter was applied. The heading angle outputs of last 100 s of the RKF were averaged to obtain the results listed in Table 1. The “Error” item in Table 1 represents the results minus the averaged value of the seven experiments. **Std** represents the standard deviation of seven experiments.

**Figure 13:**
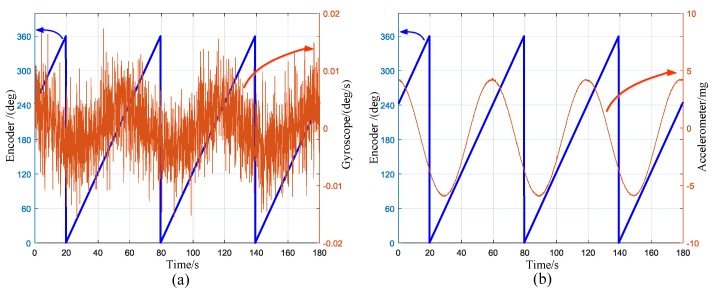
Resampled data outputs (10 Hz): (**a**) gyro and encoder; and (**b**) accelerometer and encoder.

The outputs of gyroscope and encoder are displayed in Figure 13a. The left ordinate is the encoder output while the right side is the gyro output. The data were averaged at the sample rate of 10 Hz. The sinusoid shape indicates the projection of the north component of the Earth’s rotation rate on the gyroscope input axis. The amplitude of the sine wave is around 0.003°/s, which is in good agreement with the north projection of the earth rotation rate at latitude 40°. The outputs of the accelerometer and the encoder are exhibited in Figure 13b. The left ordinate is the encoder output while the right side is the accelerometer output. The sinusoid shape indicates the attitude angle of the prototype.

**Figure 14:**
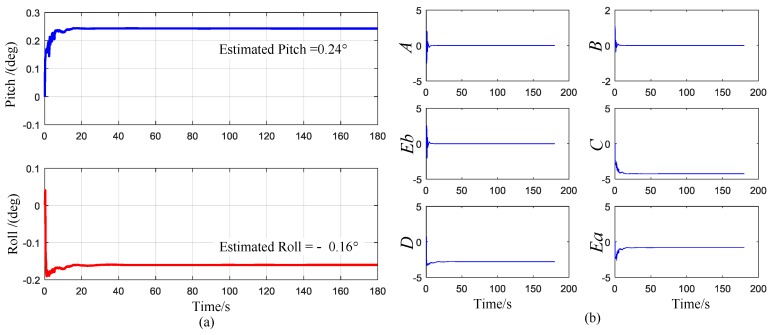
(**a**) The estimated attitude angle (**b**) The Kalman outputs of the system state vector *X*.

**Figure 15:**
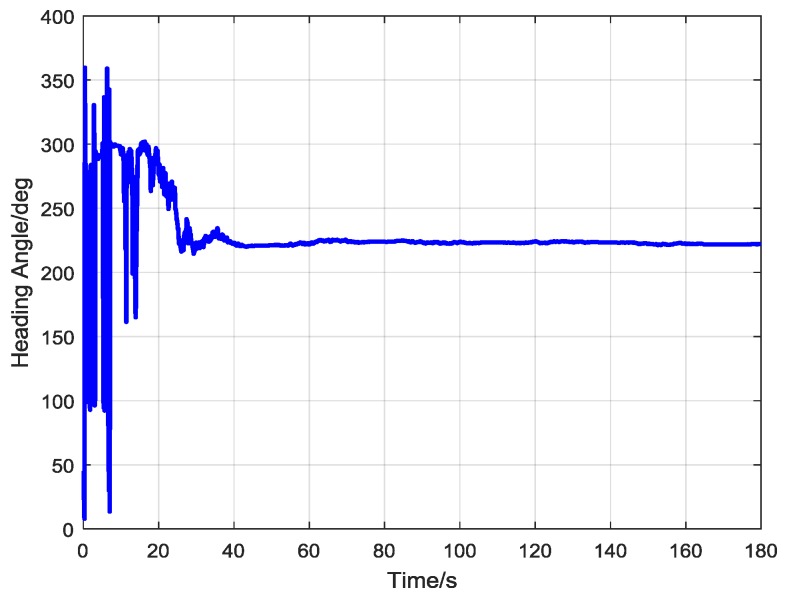
The heading angle convergence curve of the robust Kalman filter (RKF).

Figure 14a,b show the estimated attitude angle and the system vector output of the RKF, respectively. The output heading angle of the RKF during one experiment is displayed in Figure 15. Apparently, the heading angle converged to a steady value after about 40 s. The standard deviation of seven experiments is around 1° as listed in Table 1.

### 4.3. Error Analysis and Compensation

#### 4.3.1. Prototype Attitude Angle Error

**Figure 16:**
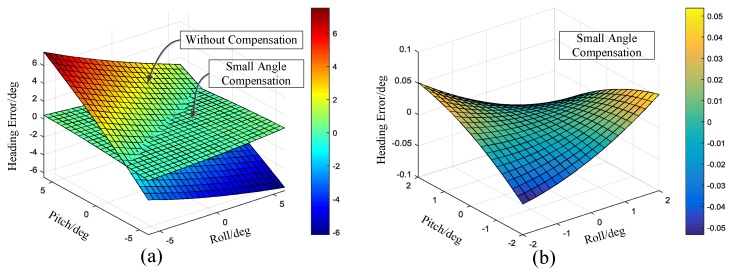
(**a**) Heading angle error without attitude compensation and with small angle compensation; (**b**) Heading angle error with small angle compensation.

From the all-attitude gyro north finding model, the attitude error will cause the vertical component of the Earth’s rotation vector to project on the gyro sensitive axis plane. This projection will form a new vector with the north component of the Earth’s rotation vector. The measured north direction will be the new synthetic vector. The attitude error will lead to the systematic deviation. Simulations are performed to study the effect of pitch angle and roll angle from −6° to 6°, respectively. The results are presented in Figure 16. In this paper, the attitude error was compensated through an accelerometer. In order to implement the compensation in embedded systems, the small angle compensation formula (Equation (29)) was used. The residual errors after small angle compensation was also shown in Figure 16. It can be seen that if the pitch and roll angle are less than 2°, the residual error will be less than 0.06°. The compensation precision can meet the requirements of the MEMS gyro north finder.

#### 4.3.2. Gyro Temperature Drift Error

Temperature drift has a great influence on the outputs of the gyroscope. The temperature drift coefficient of a MEMS gyro is usually from a few to several hundred degrees per hour per degree centigrade. A temperature drift coefficient of 1°/h/°C will lead to nearly a 5° heading angle error with the temperature rising 1 ℃. This error item should be carefully compensated.

**Figure 17:**
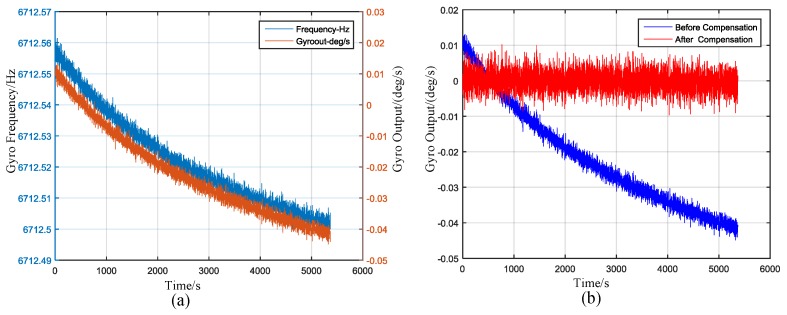
(**a**) Gyro output and gyro frequency (indicating temperature) output; (**b**) Gyro output before and after compensation.

A first-order polynomial compensation was used. The temperature output and the gyroscope output can be seen from Figure 17a. The gyro frequency indicates the temperature [33]. The compensation results can be seen from Figure 17b. The effect of temperature can be ignored after compensation.

#### 4.3.3. Gyro *g*-Sensitivity Error

Gyro *g*-sensitivity is also called gyro linear acceleration sensitivity, which means the gyro output will differ from different linear acceleration input. It is usually an important error parameter for angular sensors. For our prototype, the tilt angles (i.e., the roll angle and pitch angle) will make the gyro output change with the motor rotation angle. Suppose the gyro *g*-sensitivity on the three mutually perpendicular axes are $\;\left[k_{\mathrm{gx}}\;k_{\mathrm{gy}}\;k_{\mathrm{gz}}\right]$. Then the gyro output caused by gyro *g*-sensitivity can be calculated with the input acceleration as follows:

(33) $$W_{\mathrm{gmy}}=\left[k_{\mathrm{gx}}\;k_{\mathrm{gy}}\;k_{\mathrm{gz}}\right]{\left[f_{mx}\;f_{my}\;f_{mz}\right]}^{T}$$

where $\;k_{\mathrm{gx}}\;,k_{\mathrm{gy}},k_{\mathrm{gz}\;}\;$ denote the gyro *g*-sensitivity in its *X*, *Y* and *Z* axes, respectively. The *X* axis is the gyro input axis. The *Y* and *Z* axes are the other two perpendicular axes. To analyze the maximum errors caused by gyro *g*-sensitivity, suppose $\;k_{\mathrm{gx}}=max\left\{k_{\mathrm{gx}},k_{\mathrm{gy}}\right\}$. For small values of *γ* and *θ*, we can use the following approximations:

(34) cosθ≈1, sinθ≈θ, sinθsinγ≈0

With above approximations and the acceleration in Equation (10), Equation (33) can be reduced to:

(35) $$W_{\mathrm{gmy}}=k_{\mathrm{gx}}\left(\gamma -\theta \right)\left(\cos\alpha +\sin\alpha \right)g-k_{\mathrm{gz}}g$$

**Figure 18:**
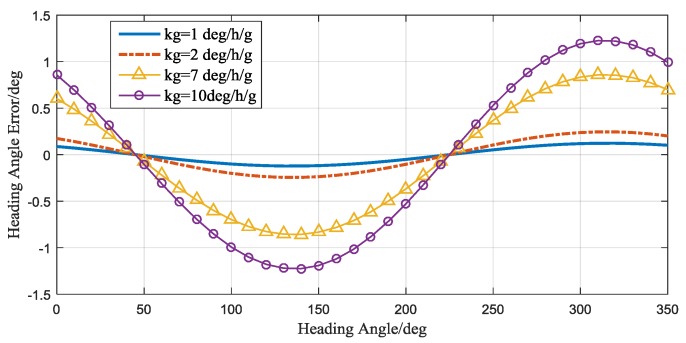
The heading angle error caused by gyro linear acceleration sensitivity error.

As can be seen in the above equation, $k_{\mathrm{gz}}$ directly influence the gyro output, $k_{\mathrm{gz}}$ have the impact through the tilt angle. Usually, the tilt angles are small angles. Then $k_{\mathrm{gz}}$ have the largest influence. Fortunately, this influence is equal to a constant bias on gyro output, and does not cause a heading error through rotation modulation. The tilt angle difference  (γ−θ)  will cause the gyro output change with the rotation angle  α. This will create a constant heading bias changing with the heading angle. When the roll angle and the pitch angle have different signs, the largest errors will be introduced. Simulating this error when the roll angle and pitch angle are 0.5° and −0.5°, respectively, the results can be seen in Figure 18.

For our north finder prototype, a custom in-house CSQMG gyroscope was made. With an in-plane dual tuning fork resonator and differential signal extraction, the common mode linear interferences such as acceleration and shock are rejected [30]. Thanks to the well-designed symmetric MEMS structure, the nominal *g*-sensitivity of the MEMS gyroscope is around 7°/h/g for the gyro input axis and 3°/h/g for the other two perpendicular axes. The maxim error caused by gyro *g*-sensitivity is less than 1 degree. Moreover, this error is a fixed error as can be seen from Equation (35). The gyro *g*-sensitivity errors can be estimated and compensated since the roll angle and pitch angle are estimated [34]. The errors caused by gyro *g*-sensitivity can be ignored.

#### 4.3.4. Motor Speed Fluctuation Error

For the static north finding scheme, the motor’s speed fluctuations will not cause heading errors since the ISA is measured in one direction, the data during the rotating process will be abandoned. For the dynamic north finding scheme, the speed fluctuation will bring out heading errors. We denote the non-orthogonal angle between the motor rotating axis and gyro input axis as  η. We also denote the motor speed as  ω, then the motor rotation angle  α=ωt. If there exists a motor speed fluctuation Δω, the gyro output will change with the speed fluctuation. The gyro output Equation (15) can be expressed as follows:

(36) $$W_{iemy}=A\sin\left(\omega +\Delta \omega \right)t+B\cos\left(\omega +\Delta \omega \right)t+\left(\omega +\Delta \omega \right)\sin\eta +E_{b}$$

As can be seen from Equation (36), the motor speed  ω will have a constant projection  ωsinη through the non-orthogonal angle  η. The projection is a constant for an assembled north finder. It is the same as the gyro bias, which also has no influence on the results. The speed fluctuation Δω will be coupled into the gyro output through the non-orthogonal angle η. The system can achieve the speed stability of 0.1–0.2% based on the former work [31]. The speed fluctuation is about 0.006–0.012°/s at the speed of 6°/s. This speed fluctuation is not directly coupled into the gyro’s output. It was introduced through the non-orthogonal angle η, which is usually less than 1 degree. It is a small constant multiplied by sinη.

**Figure 19:**
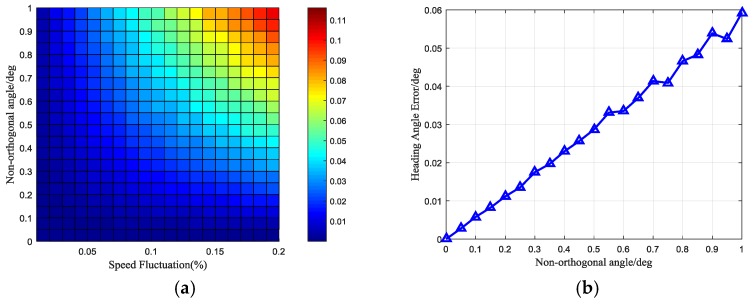
(**a**) The heading angle error caused by the motor speed fluctuation and the non-orthogonal angle; (**b**) The heading angle error when the speed fluctuation is 0.1%.

On the other hand, the speed fluctuation is a high-frequency disturbance relative to the rotation rate, and can be filtered easily. The north finding algorithm is equivalent to a band pass filter, and the speed fluctuation error can be ignored. Simulating the speed fluctuation errors using the Monte Carlo method is as follows. The rotation speed is set to 6°/s, the speed fluctuation was changed from 0.01% to 0.2% with a step of 0.01%, and the non-orthogonal angle from 0° to 1° with a step of 0.05°. 1000 simulations are conducted for each point, and then the deviation of these 1000 data was denoted as the heading error. The simulation results can be seen in Figure 19. We can see that the heading angle errors increase with the speed fluctuations and non-orthogonal angles. When the speed fluctuation is 0.1%, the heading angle error increases with the non-orthogonal angle, as can be seen in Figure 19b. The maximum heading error is less than 0.06°. This is a small error compared to the error caused by the gyro.

In our prototype, the rotation modulation technique was used to find the true north, the bias error of the gyro and accelerometer can be eliminated. They have no influence on the results. The sensitive axes of MEMS gyro and accelerometer, the rotation axis of the PMSM are designed to be perpendicular mutually in the prototype. However, there may be misalignment errors between these directions in reality. The misalignment angle between the gyro sensitive axis and rotation axis will bring a projection of the rotation speed and its fluctuations, which have been discussed above. The misalignment angle between the accelerometer the other two axes will cause the tilt compensation error. The misalignment angle is a rather tiny angle, the error caused by the tilt compensation error can be ignored compared to the other errors.

### 4.4. Results Discussion

The experimental results show that both the discrete and continuous rotation modulation can reduce or eliminate the MEMS gyro bias drift, thus improving the precision of gyroscope. Then the MEMS gyroscope can be used in the gyro compassing field. The static north finding precision is 0.66°, but the alignment time is too long for practical use. For dynamic north finding, the accuracy is a little worse than the static method, but it takes a shorter time. Dynamic north finding is a developing trend to the engineering application.

**Figure 20:**
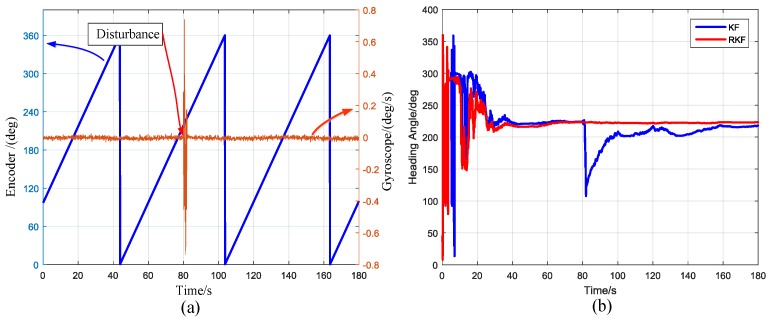
(**a**) Encoder output and Gyro output with disturbance at 80 s; (**b**) The output heading angle with ordinary Kalman filter (KF) and robust Kalman filter (RKF).

The robust performance of the north finding algorithm is not well displayed in the experimental curve because the experiment environment is better than the actual environment. The robustness of the algorithm is demonstrated when there are disturbances in the outputs of the gyro or accelerometer. Figure 20a shows such a disturbance. The disturbance was added to the gyro at 80 s, with an amplitude of 0.7°/s and a duration of 2 s. The robust Kalman filter and the ordinary Kalman filter are used for comparison. The results are exhibited in Figure 20b. For the ordinary KF, the heading angle is susceptible to interference. For the RKF, the algorithm is robust to disturbances. The RKF can suppress disturbances, and can be widely used in practical engineering applications.

The summary of current gyro north finders is shown in Table 2. Compared to the traditional gyro north finders, the MEMS gyro north finder has a lower precision at present. However, the cost of the prototype has been reduced by tens of times. Besides, the size, weight and power are also the highlights of the prototype. It bridged the gap between the low cost north finders and the small-size, low-weight and low-power man-portable devices. Moreover, the development of MEMS gyros will make the MEMS gyro north finders a very promising instrument.

## 5. Conclusions

A novel MEMS gyro north finder based on rotation modulation technique is presented in this paper. Only one single MEMS gyroscope and one single accelerometer are used in the north finder. The wireless power and wireless data transmission technique are also applied in the prototype. The use of rotation modulation technique makes it possible for the low precision MEMS gyros to be used in the north finding field. The prototype has the advantage of low cost, low size and low power. The static robust least square method (RLSM) and dynamic robust Kalman filter (RKF) north finding algorithm are analyzed and verified by the experiments. The accuracy of static experiment of the proposed north finder is 0.66°. The dynamic experiment shows the accuracy repeatability within three minutes is around 1°. The north finding precision of the prototype can already meet many application requirements. Moreover, the proposed single gyro and single accelerometer north finder structure is universal for different kinds of gyroscopes, it is valuable to both scientific research and industrial applications.

Future works will focus on the vibration and temperature experiments for practical applications. Additionally, a smaller and light-weightier MEMS gyro north finder will also be needed to meet the requirement of intelligent wearable equipment for individual solider navigation.

## Figures and Tables

**Table 1:** Dynamic north finding results.

Run	1	2	3	4	5	6	7	Std
Results	222.52°	220.95°	222.18°	223.34°	222.92°	222.94°	220.61°	1.05°
Error	0.31°	−1.26°	−0.03°	1.13°	0.71°	0.73°	−1.60°	

**Table 2:** The summary of current gyro north finders.

Name	Gyromat 3000	HG 2172	Octans 3000	SIGMA 20M	This Paper
**Producer**	DMT GmbH	Honeywell	iXBlue	SAFRAN	This Paper
**Gyros**	Mechanical	RLG	FOG	HRG	MEMS
**Time**	10 min	4 min	5 min	6 min	3 min
**Precision**	3.24″	0.05°	0.1°	0.1°	1°
**Size/mm**	Φ215 × H330	163 × 165 × 163	Φ213 × H375	208 × 136 × 292	110 × 140 × 50
**Weight**	11.5 kg	4.1 kg	15 kg	4.5 kg	1.5 kg
**Power**	Not Specified	18 W	20 W	28 W	3.6 W
**Type**	Product	Product	Product	Product	Prototype
